# Nanosensors Made of Halloysite and Kojic Acid Metal Complexes for Dopamine Detection

**DOI:** 10.1021/acsanm.5c02759

**Published:** 2025-07-31

**Authors:** Angelo Ferlazzo, Maria Teresa Armeli Iapichino, Giulia Calabrese, Giovanna D’Accurso, Roberto Fiorenza, Venerando Pistarà, Antonino Gulino, Antonio Rescifina, Vincenzo Patamia, Giuseppe Floresta

**Affiliations:** † Department of Chemical Sciences, 9298 University of Catania, Viale Andrea Doria 6, 95125 Catania, Italy; ‡ Department of Drug and Health Sciences, 9298 University of Catania, Viale Andrea Doria 6, 95125 Catania, Italy

**Keywords:** Electrochemical sensor, Nanocomposite, Dopamine, Real sample analysis, Chelating agent, Sustainable material

## Abstract

A nanocomposite sensor has been developed by integrating halloysite nanotubes (HNTs), kojic acid (K), and Cu^2+^ ions (HNTK-Cu), marking a significant advancement in the field of dopamine detection. This cutting-edge sensor leverages the synergistic properties of its components to deliver exceptional analytical performance with promising implications for biomedical diagnostics and food safety monitoring. This innovative sensor exploits the unique properties of halloysite nanotubes and kojic acid to achieve a superior performance. Among its most notable features, the HNTK-Cu sensor exhibits exceptional sensitivity, reaching a limit of detection (LOD) of as low as 68 nM, enabling the accurate quantification of even trace levels of dopamine. Furthermore, it demonstrates remarkable selectivity, effectively discriminating dopamine from structurally similar or commonly interfering substances, a crucial requirement for reliable real-world applications. The sensor also offers excellent operational stability, maintaining a consistent performance across multiple detection cycles, which is critical for long-term and repetitive reuse. From a synthetic standpoint, the fabrication of the HNTK-Cu nanocomposite is both straightforward and environmentally friendly, representing a sustainable and cost-effective alternative to conventional dopamine sensors. Notably, the HNTK-Cu sensor has demonstrated the capability to perform electrochemical detection in complex matrices, including food samples and fetal bovine serum, underscoring its immediate applicability in practical scenarios. The sensor’s superior performance arises from the unique synergy between its components: the high surface area and robust mechanical/thermal stability of halloysite nanotubes and the strong metal-chelating ability of kojic acid, which enhances both the loading and coordination of Cu^2+^ ions, critical to the sensor’s electrochemical activity.

## Graphical abstract

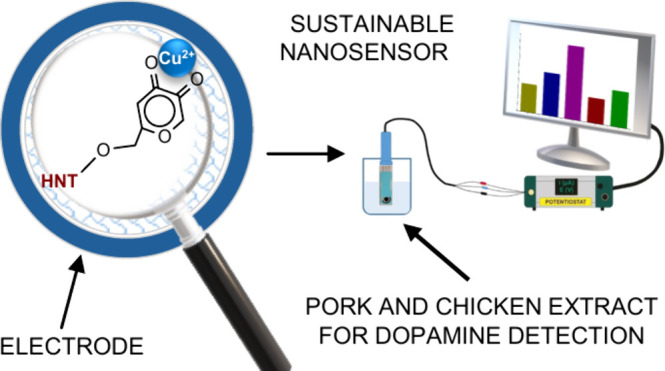

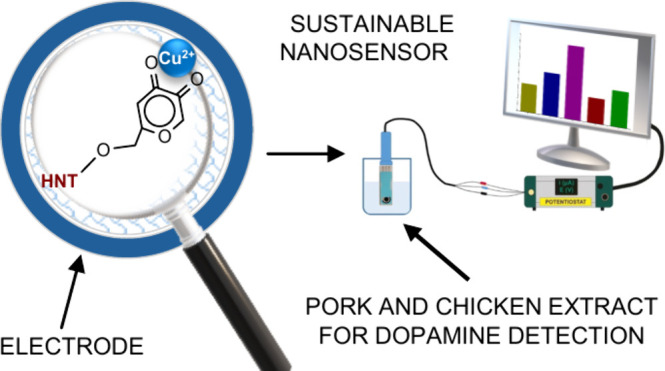

## 1 Introduction

Dopamine (4-(2-aminoethyl)­benzene-1,2-diol), an essential neurotransmitter within the catecholamine family, plays a crucial role in regulating renal hormones, the cardiovascular system, and the central nervous system. It significantly influences individuals’ physical, mental, and emotional experiences, as neurotransmitters control nearly all bodily functions.^1^ Numerous studies have shown that abnormal dopamine levels are linked to Parkinson’s disease, schizophrenia, depression, and dementia.^2^

Notably, dopamine can accelerate the breakdown of body fat and enhance protein deposition in animals, thereby improving the lean meat yield and feed conversion efficiency. Because of these effects, it has been illegally used as a “lean meat agent” in livestock farming to expedite meat production for human consumption, despite being globally banned.^3,4^ However, prolonged consumption of food containing dopamine residues can lead to adverse health effects, including palpitations, headaches, and metabolic disorders.^5^

Another key neurotransmitter, serotonin, undergoes electrochemical oxidation at potentials similar to those of dopamine due to its similar structure.^6^ Serotonin primarily regulates mood, sleep, appetite, and digestion, contributing to feelings of well-being and calm. Imbalances in either neurotransmitter can have a significant impact on mental and physical health.^7^

To prevent these illegal practices and ensure food safety, it is paramount to develop highly sensitive, eco-friendly, and sustainable sensor technologies for dopamine detection. Advancing innovative, eco-friendly, and cost-effective detection methods will strengthen regulatory enforcement and promote a safer, more environmentally responsible approach to food production.

Dopamine sensing technology has also significantly advanced through electrode modifications in electrochemical sensors, utilizing enzymes, aptamers, inorganic materials, and molecularly imprinted polymers (MIPs).^8^ Among these, enzyme-based sensors offer high sensitivity and specificity but suffer from poor stability in extreme conditions.^9^ Aptamer-based sensors are more resilient but still require stabilizing materials to maintain performance.^10^ Inorganic materials, such as graphene and metal nanoparticles, enable high sensitivity but are prone to interference and instability.^11^ Lastly, MIP-based sensors offer greater environmental stability while exhibiting lower affinity and susceptibility to cross-reactivity.^12^

Future research aims to enhance stability, specificity, and sensitivity through the integration of nanomaterials and improved fabrication techniques, with a focus on developing noninvasive, high-performance dopamine sensors. In this context, the proper modification of electrodes for electrochemical detection, whether amperometric or voltammetric, is essential for many analytes. It is within this crucial context that our work is developed.^13–15^

To address the challenges associated with developing more efficient, easily synthesized, and environmentally sustainable systems, we investigated a material based on halloysite (Al_2_Si_2_O_5_(OH)_4_·2H_2_O)^16^ and kojic acid (5-hydroxy-2-(hydroxymethyl)-4*H*-pyran-4-one), recently developed by our research group,^17–19^ as a sensor for dopamine detection. This material is composed of naturally derived starting materials, halloysite and kojic acid, and can address the aforementioned issues. It can be easily synthesized through a rapid process, without requiring drastic reaction conditions.^14^

Additionally, the combination of halloysite nanotubes (HNTs)—with a diameter between 30 and 70 nm and a length between 1 and 3 μm^20–22^ —and kojic acid^23–25^ provides key advantages. Halloysite contributes mechanical and thermal stability as well as a high surface area, while kojic acid offers chelating properties that allow for the retention of metals necessary for dopamine detection. Taking advantage of these chelating properties, particularly for bivalent metals, namely Cu­(II), Ni­(II), and Zn­(II),^24^ we decided to synthesize three complexes with these three bivalent metals. These are, among other things, also the most commonly used metals for the electrochemical detection of dopamine.^26–34^

We have achieved a strong synergy for effective dopamine sensing in real-world matrices like meat and bovine serum, using a low amount of Cu­(II) ions. The development of the halloysite nanotube (HNT), kojic acid (K), and Cu^2+^ ion nanosensor (HNTK-Cu) directly addresses the urgent need for sustainable and efficient analytical tools, particularly for dopamine detection. Its sustainability and cost-effectiveness stem from the wide availability and low cost of its main components: halloysite and kojic acid. This not only makes it eco-friendly but also ideal for large-scale production. Moreover, the HNTK-Cu sensor successfully tackled the efficiency challenge. Preliminary results indicate significant improvements in both sensitivity and selectivity, demonstrating excellent performance even in complex matrices, such as meat and bovine serum. This robustness in real-world samples underscores the material’s potential as a viable and efficient alternative to existing dopamine sensors. In essence, the HNTK-Cu nanosensor offers a powerful combination of sustainability, cost-effectiveness, and superior analytical performance, serving as a prime example of how innovative material science can meet the demand for greener and more effective solutions.

## 2 Materials

### 2.1 General Information

All chemicals were purchased from Merck and VWR: halloysite (HNT, cat. no. 685445), kojic acid (purity ≥98.5%), triethylamine (Et_3_N, ≥99%), CuCl_2_ (powder, 99%), ZnCl_2_ (powder, ≥99.995%), and Ni­(OCOCH_3_)_2_·4H_2_O (powder, 98%). Precoated aluminum sheets (silica gel 60 F254, Merck) were used for thin-layer chromatography (TLC), and the plates were visualized under UV light. ^1^H NMR spectra were recorded at 300 K on a Varian UNITY Inova spectrometer (500 MHz), using CDCl_3_ as the solvent. Chemical shift (δ) values are given in ppm.

### 2.2 Synthesis of Materials

#### 2.2.1 General Procedure for the Synthesis of HNTK Sensor

This optimized method slightly differs from that reported in our previous studies.^18,19,35^ Halloysite (HNT) (100 mg) was suspended in a 10 mL round-bottomed reaction flask containing 5 mL of dry cyclopentyl methyl ether (CPME). Et_3_N (1.56 mL, 3 equiv, 11.2 mmol) was added, and the mixture was stirred at room temperature for 1 h. Subsequently, chlorokojic acid (K) (600 mg, 1 equiv, 3.73 mmol) was added, and the reaction mixture was stirred for 24 h at 80 °C. The resulting precipitate was isolated by centrifugation, thoroughly washed with H_2_O (5 × 10 mL), and then dried at 80 °C overnight, yielding 340 mg of pure product.

#### 2.2.2 General Procedure for the Synthesis of HNTK-M

To a dispersion of HNTK (100 mg) in H_2_O (8 mL) was added metal (M), CuCl_2_ (50 mg), ZnCl_2_ (50 mg), or Ni­(OCOCH_3_)_2_·4H_2_O (50 mg).^20^ The resulting mixture was stirred at 55 °C overnight. The solvent was then evaporated under reduced pressure to yield the product, which was washed and centrifuged multiple times with deionized H_2_O to eliminate the excess uncomplexed metal ions.

### 2.3 Characterization

#### 2.3.1 Infrared Spectroscopy

Attenuated total reflectance-Fourier transform infrared spectroscopy (FTIR-ATR) analyses were conducted using an FTIR Agilent Cary 630 instrument equipped with an ATR sampling module. Thin films of the samples were applied to the ATR crystals and pressed gently. The results were derived from 512 scans acquired in the 4000–500 cm^–1^ range with a resolution of 2 cm^–1^ at room temperature.

#### 2.3.2 Thermogravimetric Analysis

Thermal gravimetric analysis (TGA) was used to study the thermal behavior under 1 atm of prepurified nitrogen, with a heating rate of 10 °C/min, in the temperature range of 50–900 °C.

#### 2.3.3 UV–Vis DRS Analysis

The ultraviolet–visible diffuse reflection (UV–vis DRS) measurements were performed using a Jasco V-670 instrument equipped with an integrating sphere, with BaSO_4_ as the reference.

#### 2.3.4 Surface Area (BET)

The Brunauer–Emmett–Teller (BET) surface area of the samples was determined through N_2_ physisorption measurements at −196 °C using a Micromeritics ASAP 2020 instrument. The samples were pretreated with an outgassing step at 80 °C.^36,37^

#### 2.3.5 X-ray Photoelectron Spectra (XPS)

X-ray photoelectron spectra (XPS) were measured at a 45° takeoff angle relative to the surface sample holder with a PHI 5000 Versa Probe II system (ULVAC-PHI, INC., base pressure of the main chamber 1 × 10^–8^ Pa).^38,39^ Samples were excited with the monochromated Al Kα X-ray radiation using a pass energy of 5.85 eV. The instrumental energy resolution was ≤0.5 eV. The XPS peak intensities were obtained after Shirley’s background removal.^38,39^ Spectra calibration was achieved by fixing the Ag 3d_5/2_ peak of a clean sample at 368.3 eV.^40,41^ The atomic concentration analysis was performed by considering the relevant atomic sensitivity factors. Some XP spectra were fitted using XPSPEAK4.1 software by fitting the spectral profiles with Gaussian envelopes after subtraction of the background. This process involves data refinement using the least-squares fitting method until the highest possible correlation between the experimental spectrum and the theoretical profile is achieved. The residual or agreement factor *R*, defined by *R* = [∑(*F*_obs_ – *F*_calc_)^2^/∑(*F*_obs_)^2^]^0.5^, after minimization of the function ∑(*F*_obs_ – *F*_calc_)^2^, converged to the value of 0.03.

#### 2.3.6 Evaluation of Mean Particle Size and Polydispersity Index

To evaluate the mean particle size (Z-ave) and polydispersity index (PdI) of HNT, HNTK, and HNTK-M­(Cu, Ni, and Zn), they were solubilized/suspended in water (1 mg/mL) and analyzed by using Photon Correlation Spectroscopy (PCS) with a Zetasizer Nano S90 instrument (Malvern Instruments, Malvern, UK). The instrument was set with a detection angle of 90° and a 4 mW He–Ne laser operating at 633 nm at a temperature of 25 °C. Three sets of measurements were used in the sample analysis, and the mean size ± standard deviation (SD) was reported as the result.

#### 2.3.7 Electrochemical Measurements

Cyclic voltammetry (CV), electrical impedance spectroscopy (EIS), differential pulse voltammetry (DPV), and chronoamperometric (CA) measurements were performed using a DropSens μStat-i 400s potentiostat/galvanostat equipped with Dropview 8400 software. A 0.1 M phosphate-buffered saline (PBS) solution with a pH of 7.4 was used for electrochemical measurements. CV was performed at a scan rate of 50 mV/s in the −0.4 to 1.1 V potential range using PBS, 10 mM potassium ferricyanide (K_3_[Fe­(CN)_6_]), and 0.1 M KCl standard solutions. EIS tests were conducted using 10 mM potassium ferricyanide (K_3_[Fe­(CN)_6_]) and 0.1 M KCl standard solutions in the 0.1–1.0 Hz frequency range, amplitude 10 mV, and applied potential 0.25 V.

DPV tests were conducted using an optimized potential step (*E*_step_) of 0.03 V, potential pulse (*E*_puls_) of 0.09 V, and time pulse (*T*_pul_) of 200 ms, with a scan rate of 40 mV/s in the −0.3 to 1.1 V potential range. CA curves were obtained by recording the oxidation current at a constant potential of 0.3 V and using CV analysis, under the same conditions as described above. At the same time, an appropriate volume of 10 mM dopamine solution was added to the electrolyte solution (PBS 0.1 M) under magnetic stirring.

Measurements were made using a commercial reference Screen-Printed Carbon Electrode (SPCE), from the Metrohm DropSens company, and a working SPCE modified with HNTK-M (M = Ni, Zn, Cu; hereafter HNTK-M/SPCE) by depositing 10 μL of a suspension of HNTK-M (5 mg in 1 mL of distilled water).

The resulting sensor was air-dried at room temperature for 24 h (Scheme 1). The sensor sensitivity (*S*) was always calculated (eq 1) as the ratio between the slope (*m*) of the calibration line and the geometric surface area (*A*) of the SPCE electrode (0.125 cm^2^).^42^ The LOD was calculated by multiplying the ratio between the standard error value of the intercept (SE_int_) and the slope (*m*) of the calibration line by 3.3 (eq 2).^43^

$$S=\frac{m}{A}$$ 1

$$LOD=3.3\times \frac{SE_{int}}{m}$$ 2

**Scheme 1:**
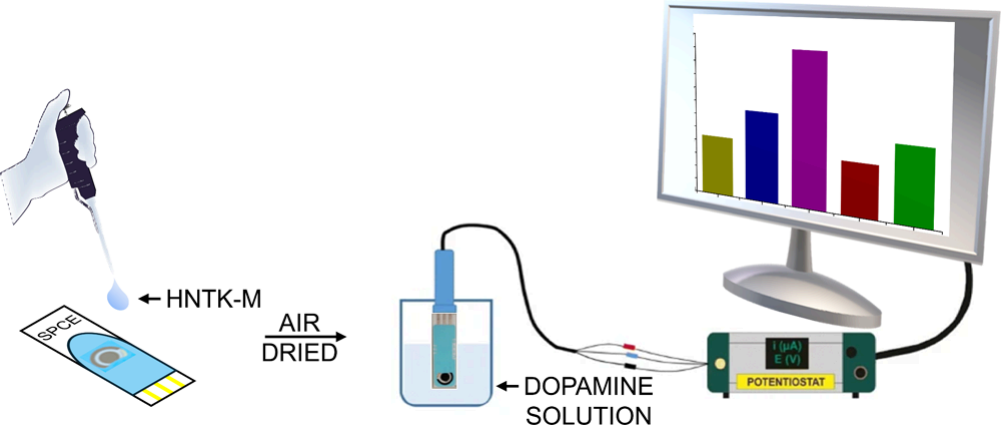
Schematic Setup of Electrochemical Experiments for Testing Halloysite Nanotubes (HNT), Kojic Acid (K), and M^2+^ Ions (Cu, Ni, or Zn; HNTK-M)

#### 2.3.8 Electrochemical Measurements for Fetal Bovine Serum and Real Meat Samples

To investigate the ability of the HNTK-Cu/SPCE sensor (vide infra) to detect dopamine in real samples, we performed the DPV analysis on fetal bovine serum (FBS) before and after spiking with 1, 7, and 15 μM dopamine, using the addition method.^3,44^ The starting dopamine concentration in the serum sample can be described as the *x*-intercept in the regression equation and was calculated using the formula

$$x_{int}=\frac{C_{a}\times V_{0}}{C_{s}}$$ 3

where *C*_a_ is the concentration of added dopamine in the serum sample, *V*_0_ is the volume of the serum sample, and *C*_s_ is the concentration of dopamine in the stock solution.^45,46^

Additionally, we investigated the dopamine concentration in commercial meat (pork and chicken, obtained from a local supermarket in Catania, Italy) using DPV, with already optimized potential step, potential pulse, time pulse, and scan rate at pH 7.4. The meat extract was pretreated to remove peptide interferents, as described in a procedure reported in the literature.^4,47^ In addition, all real samples (meat extract and FBS) were diluted 30 times with 0.1 M PBS before measurements, according to a protocol already reported for this type of investigation.^23,47^

## 3 Results and Discussion

### 3.1 Synthesis

This work aims to utilize a sustainable and ecobiocompatible nanomaterial, based on natural origin materials and easily prepared, for the development of an efficient and durable nanosensor for dopamine detection. This material consists of a clay matrix (HNT) functionalized with a natural K agent for the chelation of bivalent ions such as copper (Cu^2+^), zinc (Zn^2+^), and nickel (Ni^2+^).^19,24^ Scheme 2 shows the green synthesis of HNTK performed using the green solvent CPME,^48^ followed by complexation in water to obtain HNTK-M (Cu, Ni, and Zn).

**Scheme 2:**
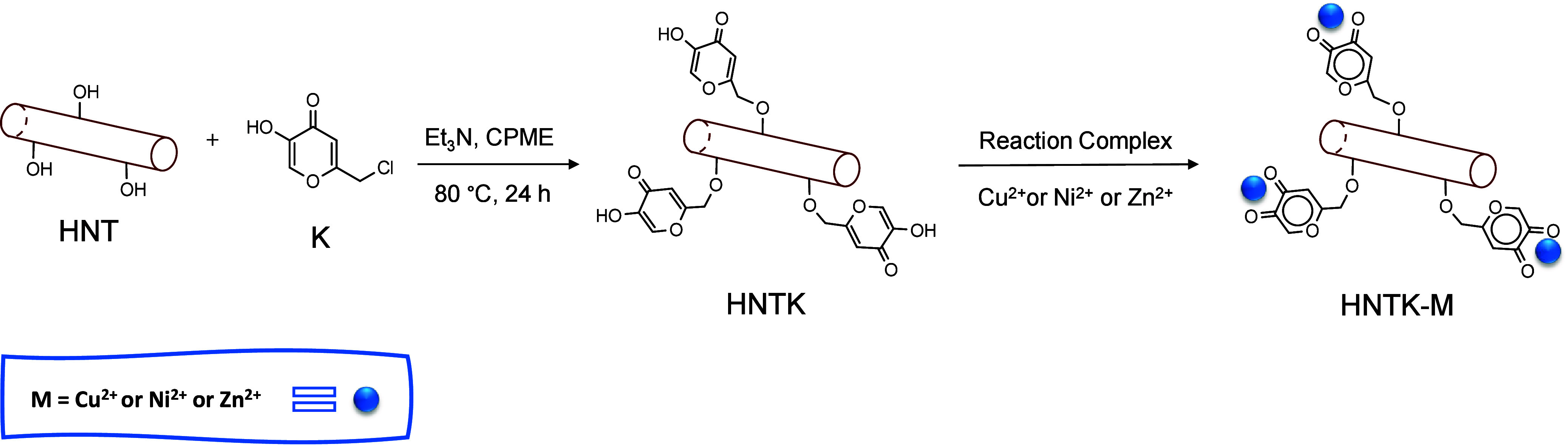
Preparation Scheme of Halloysite Nanotubes (HNT), Kojic Acid (K), and M^2+^ Ions (Cu, Ni, or Zn; HNTK-M)

### 3.2 Characterizations

The successful functionalization was confirmed by FT-IR analysis. Figure 1a displays the FT-IR spectra of HNT (green line), HNTK (black line), and HNTK-M (Cu, Ni, and Zn), demonstrating the effective modification of HNTs with K. In addition to the characteristic signals of HNTs,^21,49^ the typical bands associated with K are also present: the CH_2_ stretching vibrations at 2982 and 3069 cm^–1^, a strong signal corresponding to the C=O conjugated ketone at 1651 cm^–1^, the C=C stretching mode characteristic of an unsaturated ketone at 1620 cm^–1^, and the C–O stretching band related to kojic acid at 1215 cm^–1^.^18^ Furthermore, the presence of the metal has led to the appearance of two new bands at 1565 and 1517 cm^–1^, confirming the coordination of the carbonyl group to the metal ions^50^ (Figure 1b, red, blue, and purple lines).

**Figure 1:**
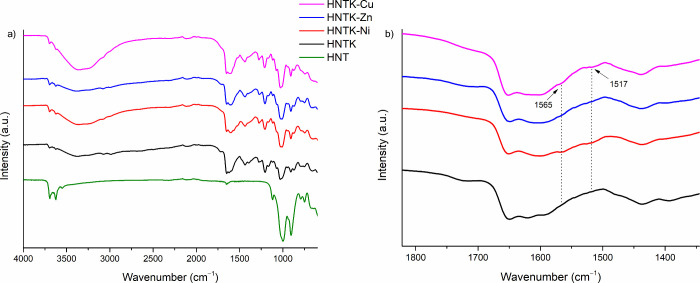
(a) Stacked FT-IR spectra of HNTK (black line), HNTK-Ni (red line), HNTK-Cu (blue line), and HNTK-Zn (purple line). (b) Expansion of the region 1600–1500 cm^–1^.

Figure S1 shows the UV-DRS spectra of the examined compounds. All samples show a broad absorption band around 250–300 nm, attributed to the chromophoric C=O group of chlorokojic acid.^51,52^ Moreover, a small feature is observable at about 600–700 nm for the HNT-Cu sample, associated with the localized surface plasmon resonance effect of copper nanoparticles.^53,54^

The degree of functionalization (%*f*) and the metal amount in each complex were calculated by thermogravimetric analyses, as reported in our previous work.^19,35^ Overlaid thermograms of the HNTK and HNTK-M series are shown in Figure 2, from which it can be seen that the presence of metals increases the residue at 900 °C.

**Figure 2:**
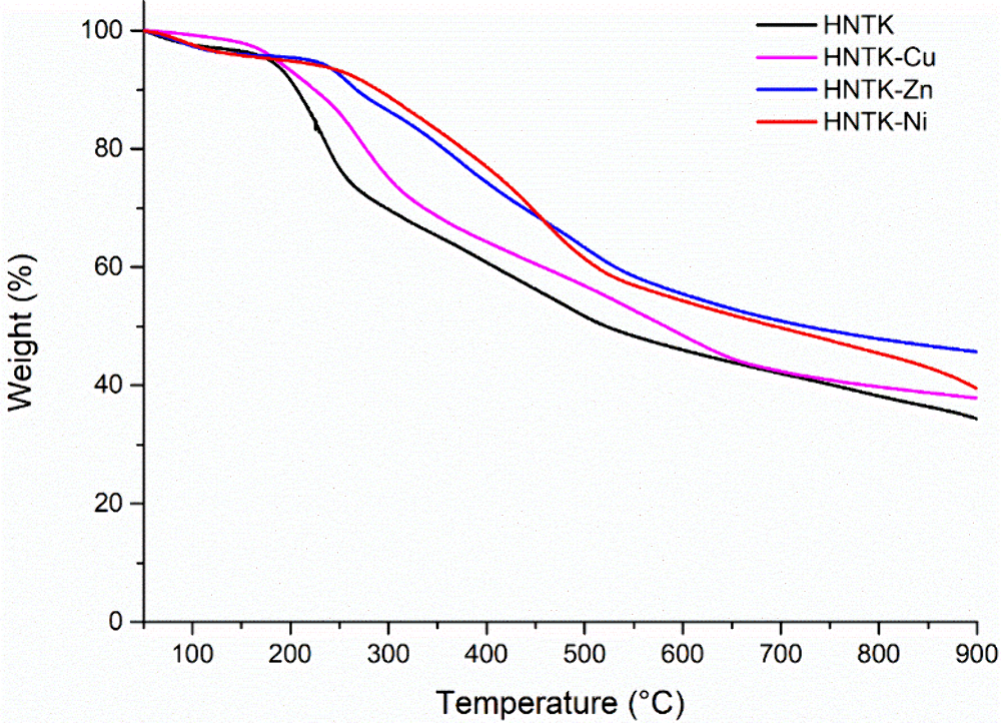
Thermogravimetric curves of the HNTK and HNTK-M series.

Table 1 shows the weight loss percentages of pristine HNT, HNTK, and HNTK-M series with various metals. The degree of functionalization (%*f*) for HNTK was calculated using eq 4, where *x* is the mass loss between 150 and 550 °C, %*f* = 39.0.

$$\%f=\frac{x_{adduct}-x_{\mathrm{HNT}}}{x_{K}-x_{\mathrm{HNT}}}\times 100$$ 4

**Table 1:** Mass Loss Percentages of Pristine HNT, K, and HNTK-M Series

	Mass loss/%					
Sample	*T* < 150 °C	150 °C < *T* < 350 °C	350 °C < *T* < 550 °C	550 °C < *T* < 900 °C	Residue/%	Amount of metal/%
HNT	1.1	1.8	12.3	1.1	83.7	
K	1.8	98.2				
HNTK	3.6	30.7	16.2	13.3	36.2	
HNTK-Cu	2.5	29.4	15.4	14.4	38.3	2.1
HNTK-Ni	4.2	12.8	24.8	16.6	41.6	3.3
HNTK-Zn	4.1	15.9	21.8	12.4	45.8	9.6

Morphological characterization was reported in our previous works and is therefore not shown here.^18,19,35^

The size of water-suspended aggregates of HNT, HNTK, and HNTK-M (Cu, Ni, and Zn) was analyzed with PCS (Table 2). The results confirmed that HNT exhibited a size in the micrometer range (1103 nm), consistent with literature reports.^55^ Functionalization with K led to an increase in size (3728 nm) and a decrease in polydispersity (from 0.598 to 0.508). The presence of metals decreases the size of the material, emphasizing the chelation by kojic acid.^56^

**Table 2:** Mean Size (Z-ave) and Polydispersity Index (PdI) of HNT, HNTK, and HNTK-M (Cu, Ni, and Zn)

Sample	Z-ave (nm) ± SD	PDI ± SD
HNT	1103 ± 131.2	0.598 ± 0.20
HNTK	3782 ± 460.6	0.508 ± 0.22
HNTK-Cu	1464 ± 464.8	0.128 ± 0.083
HNTK-Ni	1658 ± 234.4	1.0 ± 0
HNTK-Zn	761.3 ± 54.62	0.741 ± 0.449

In Figure 3, the experimental N_2_ isotherm curves of the examined samples are reported. All of them showed a type IV isotherm with an H_2_ hysteresis loop related to pores with wide bodies and narrow necks.^37^ The commercial halloysite (HNT, cat. no. 685445) exhibited a BET surface area value of 64.9 m^2^/g according to the supplier’s data sheets. The K functionalization increased the surface area (68.2 m^2^/g for the HNTK sample), whereas the addition of metals led to a slight variation in the BET values (Figure 3), within the range of the experimental error (±1 m^2^/g). The HNT-Cu sample presented a slightly intense H2 hysteresis loop.

**Figure 3:**
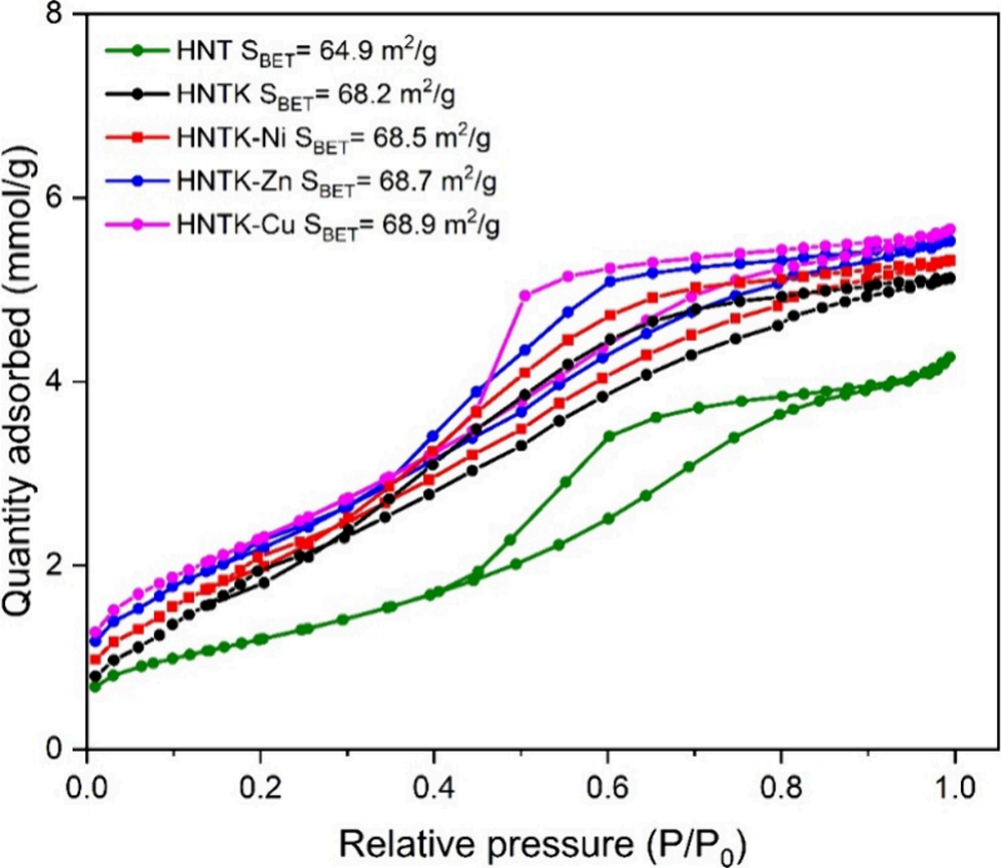
N_2_ isotherm curves of the examined samples for evaluating the BET surface area (*S*_BET_).

Some XP spectra of HNTK-Cu are reported in Figure S2, which shows the Al 2p, Si 2p, O 1s, and C 1s signals, as expected from the composition of the halloysite functionalized with kojic acid.^57^

The XP spectrum of Cu 2p states confirms copper complexation with HNTK. A careful deconvolution of the experimental spectrum required three Gaussian doublets at 932.8–952.6 eV due to the Cu 2p_3/2_/2p_1/2_ spin–orbit pair, strongly indicative of the presence of Cu^0^ states (relative intensity 68%),^58^ 935.3–955.1 eV due to the Cu 2p_3/2_/2p_1/2_ spin–orbit couple due to the Cu^2+^ states (relative intensity 32%), and a satellite peak at 943.6 eV confirming the presence of Cu^2+^ (vide infra).

### 3.3 Sensor Activity

We studied the sensing capabilities of bare SPCE and HNT-, HNTK-, and HNTK-M (M = Ni, Zn, Cu)-modified SPCE using CV analysis in the presence of 100 mM dopamine. Figure 4 shows that the HNTK-Cu-modified sensor yielded a better response, evaluated by considering the maximum anodic oxidation peak (*I*_p,ox_) at approximately 0.2 V, compared to those of the others. The observed response for HNTK-Cu is about three times as great as for SPCE and the other modified electrodes (HNT/SPCE, HNTK-Ni/SPCE, HNTK-Zn/SPCE), and twice as great as for HNTK/SPCE. Therefore, given its superior performance, we will refer to results obtained using HNTK-Cu/SPCE from here onward.

**Figure 4:**
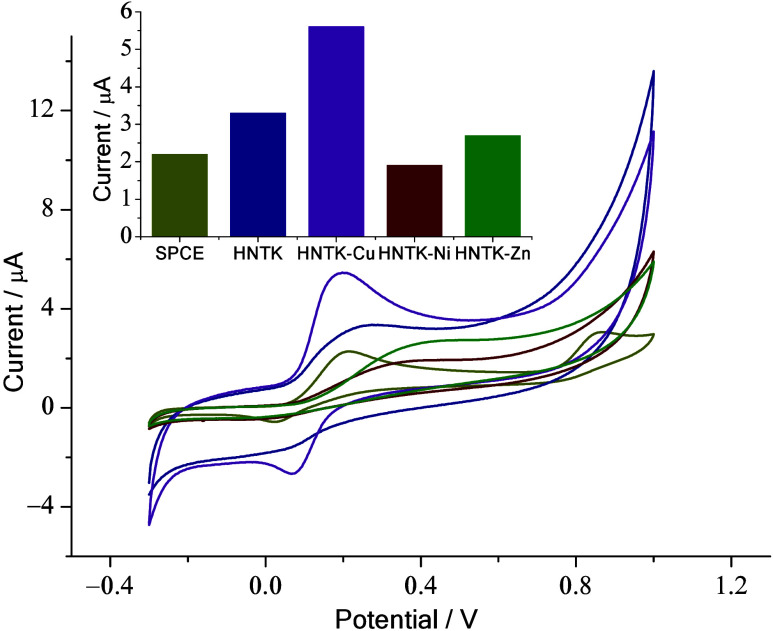
Electrochemical behavior of SPCE, HNT/SPCE, HNTK/SPCE, HNTK-Cu/SPCE, HNTK-Ni/SPCE, and HNTK-Zn/SPCE (−0.3–1.0 V potential range) with 100 μM of dopamine. A comparison between the responses is given in the inset.

Regarding the optimization of the pH for carrying out the sensing measurements, we conducted tests at various pH values (3.0, 4.5, 5.0, 6.0, 7.4, 8.0, and 9.0). Figure S3a shows that the 100 μM dopamine *I*_pa_ values, obtained with the HNT-Cu/SPCE sensor, increase with an increase in pH, reaching a maximum value at pH 7.4. Moreover, Figure S3b shows the effect of different HNTK-Cu loadings on SPCE electrodes, and the highest dopamine *I*_pa_ was observed when 50 μg of HNTK-Cu was deposited on HNT-Cu/SPCE.^59^

HNTK-Cu/SPCE was then characterized by studying its properties using CV and EIS in 0.1 M PBS, in the presence of a standard (10 mM K_3_[Fe­(CN)_6_]). The modified sensor shows (Figures 5a–e) a CV cycle larger than that of the bare SPCE, thus indicating a larger surface area.^60^ The calculation of the electrochemically active surface area (ECSA),^61,62^ determined using the double-layer capacitance (CDL), in a non-Faradaic potential range (0.2 V), for HNTK-Cu/SPCE and SPCE shows values of 1.35 and 0.46 μF/cm^2^, respectively. The increase in ECSA is in agreement with what is observed in Figure 5b, where the CV conducted in the presence of [Fe­(CN)_6_]^3–/4–^ shows oxidation (*I*_p,ox_) and reduction (*I*_p,red_) peak intensities for HNTK-Cu higher than those for SPCE, but more importantly a decrease in peak-to-peak separation (Δ*V*) from 0.75 to 0.26 for SPCE and HNTK-Cu, respectively. These results, observed in the iron ion redox reaction, indicate that HNTK-Cu enhances the electrode’s electrochemical performance of the electrode by increasing the charge transfer and improving the electrocatalytic activity.^63^

**Figure 5:**
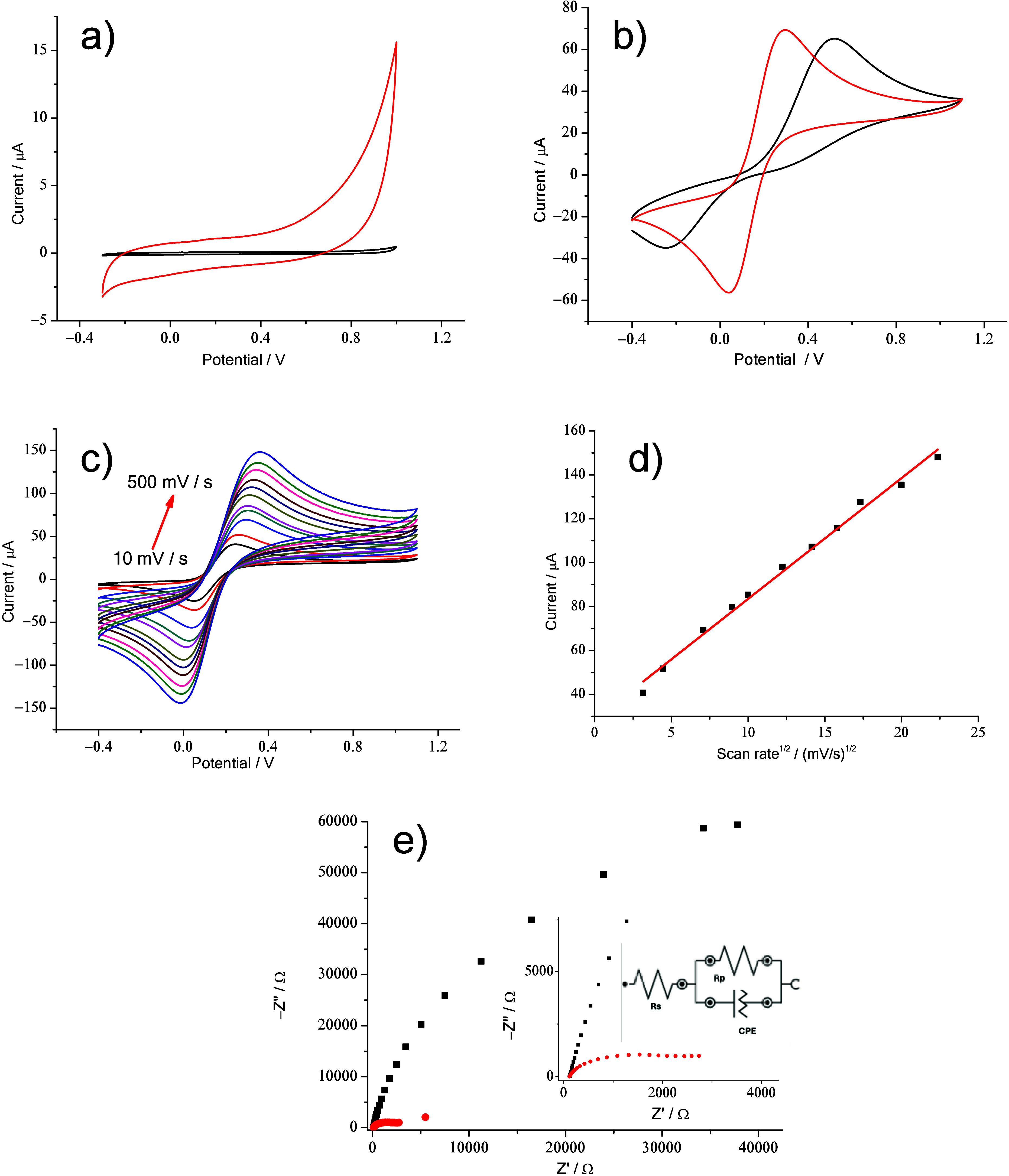
(a) Electrochemical behavior in 0.1 M PBS at a scan rate of 50 mV/s of SPCE (black line) and HNTK-Cu/SPCE (red line), in the–0.3 to 1.0 V potential window. (b) CV of SPCE (black line) and HNTK-Cu/SPCE (red line) in the presence of 10 mM K_3_[Fe­(CN)_6_] and 0.1 M PBS at a 50 mV/s scan rate, in the −0.4 to 1.1 V potential window. (c) CV of HNTK-Cu/SPCE in the presence of 10 mM K_3_[Fe­(CN)_6_] at different scan rates from 10 to 500 mV/s in 0.1 M PBS (pH 7.4). (d) Plot of *I*_p,ox_ vs υ^1/2^ (*y* = *a* + *bx*; Adj. R-Square = 0.99249; Intercept = 28.49547 ± 2.07064; Slope = 5.50173 ± 0.15131). (e) Nyquist plots of SPCE (black squares) and HNTK-Cu/SPCE (red dots), with a zoom of high frequencies and equivalent circuit in the inset.

In addition, CVs obtained in the presence of 10 mM K_3_[Fe­(CN)_6_] by varying the scan rate (from 10 to 500 mV s^–1^) show an increase in the anodic oxidation peak (*I*_p,ox_) linear to the root of the scan rate, thus highlighting a diffusive mechanism at the electrode interface (Figure 5c,d).^64^

Finally, the EIS analysis for SPCE and HNTK-Cu/SPCE produced a Nyquist diagram (Figure 5e), where a small semicircle for the modified electrode compared to the bare one shows a lower resistance in promoting the ferro/ferricyanide redox reaction; in fact, the charge transfer resistances (*R*_ct_) were 2763 and 171832 Ω for HNTK-Cu/SPCE and SPCE, respectively.

The detection capabilities of HNTK-Cu/SPCE were examined by DPV measurements (Figure 6a) using increasing concentrations of dopamine. Figure 6b shows the related calibration curve. This sensor shows a sensitivity of 2.165 μA μM^–1^ cm^–2^ and an LOD of 0.068 μM, thus evidencing an excellent responsiveness to dopamine.

**Figure 6:**
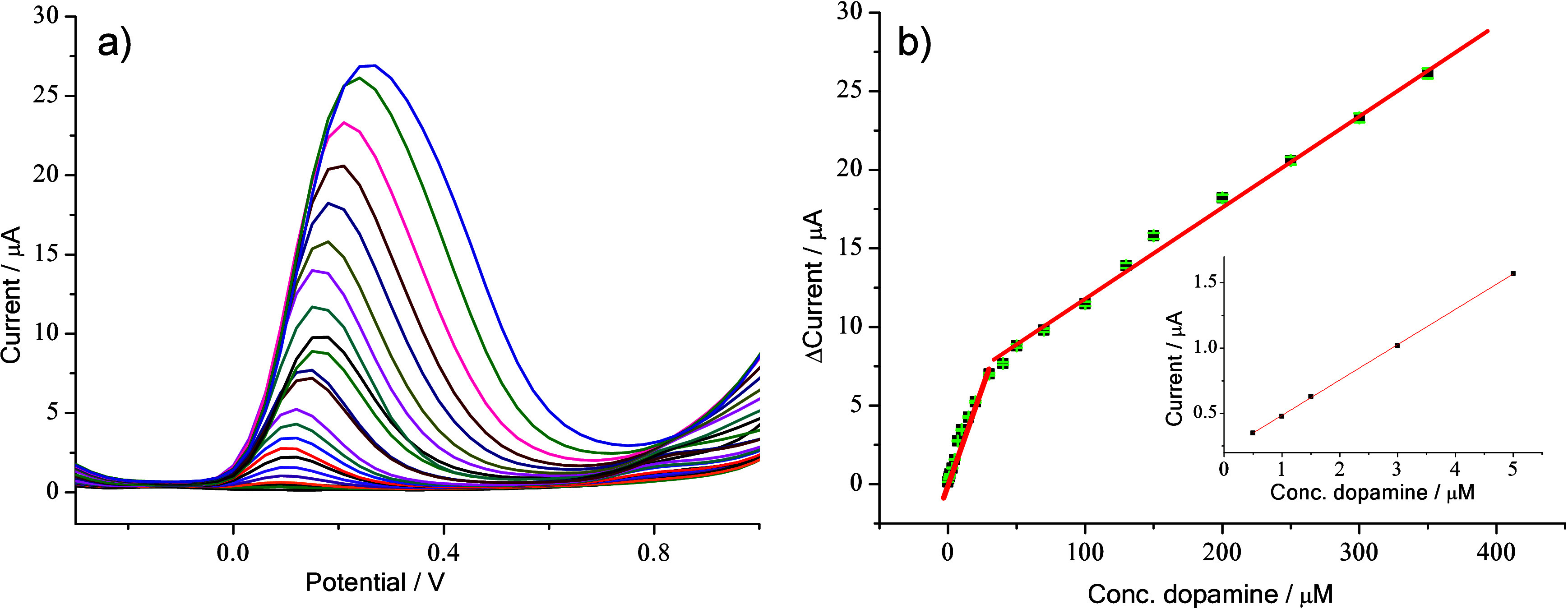
(a) DPV at different dopamine concentrations (0–350 μM, initial step 1 μM) in 0.1 M PBS (pH 7.4). (b) Calibration curve for anodic peak current (*I*_pa_) vs the dopamine concentration (RSD ≤ 1.2 for five repeated whole cycles). Inset: expanded scale in the 0–5 μM range of dopamine (*y* = *a* + *bx*; Adj. R-Square = 0.99978; Intercept = 0.21451 ± 0.005552; Slope = 0.27068 ± 0.00203).

The sensor’s performance was also investigated by chronoamperometry, and Figure 7a shows the HNTK-Cu/SPCE current response as a function of dopamine concentration (using a constant applied potential of 0.3 V vs. Ag/AgCl). The corresponding calibration curve (Figure 7b) shows the linear trend of current versus dopamine concentration (μM), yielding a sensitivity of 183 μA mM^–1^ cm^–2^ and an LOD value of 0.75 μM.

**Figure 7:**
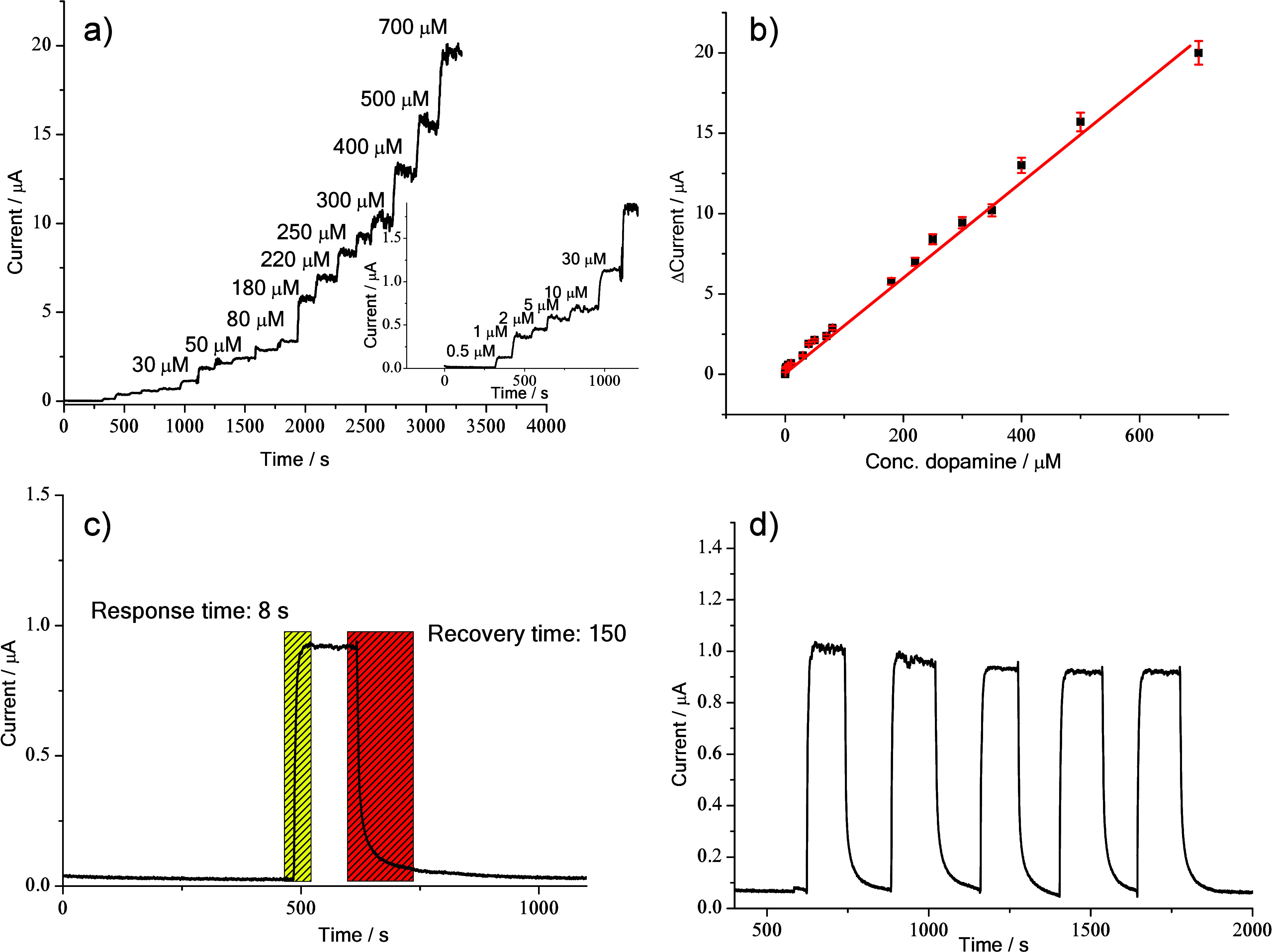
(a) Current–time response of the HNTK-Cu/SPCE electrode upon successive additions of dopamine to the 0.1 M PBS electrolyte at 0.3 V. The inset shows the response in the 0–30 μM dopamine range (*y* = *a* + *bx*; Adj. R-Square = 0.87089; Intercept = 0.032 ± 0.05697; Slope = 0.22914 ± 0.04972). (b) Calibration line for detecting and quantifying dopamine. The inset shows the calibration line in the 0–5 μM dopamine range. (c) Response and recovery time of HNTK-Cu/SPCE in the absence and presence of dopamine. (d) Repeatability test toward 30 μM of dopamine.

The response time for dopamine concentrations in the 0–700 μM range was 8 s, and the recovery of initial conditions (absence of dopamine) was 150 s (Figure 7c). These values demonstrate the excellent ability of HNTK-Cu/SPCE to work continuously over several cycles with excellent repeatability (Figure 7d; RSD value of 3.7%).

In addition, five freshly prepared HNTK-Cu/SPCE electrodes were used to measure 30 μM dopamine in 0.1 M PBS. All five electrodes showed identical DPV and CA responses, with an RSD of 2.3%, confirming the high reproducibility of the HNTK-Cu/SPCE.

We also investigated the HNTK-Cu/SPCE sensor’s ability to detect dopamine in the presence of interferents present in real samples, such as phenylalanine (Phe), tyrosine (Tyr), glucose (Glu), tyramine (Tyra), uric acid (U.A.), and ascorbic acid (A.A.) (Figure 8a). Chronoamperometry measurements reported in Figure 8 clearly show that HNTK-Cu/SPCE responds to dopamine (40 μM) and shows no change in current after the addition of 200 μM of each interferent.

**Figure 8:**
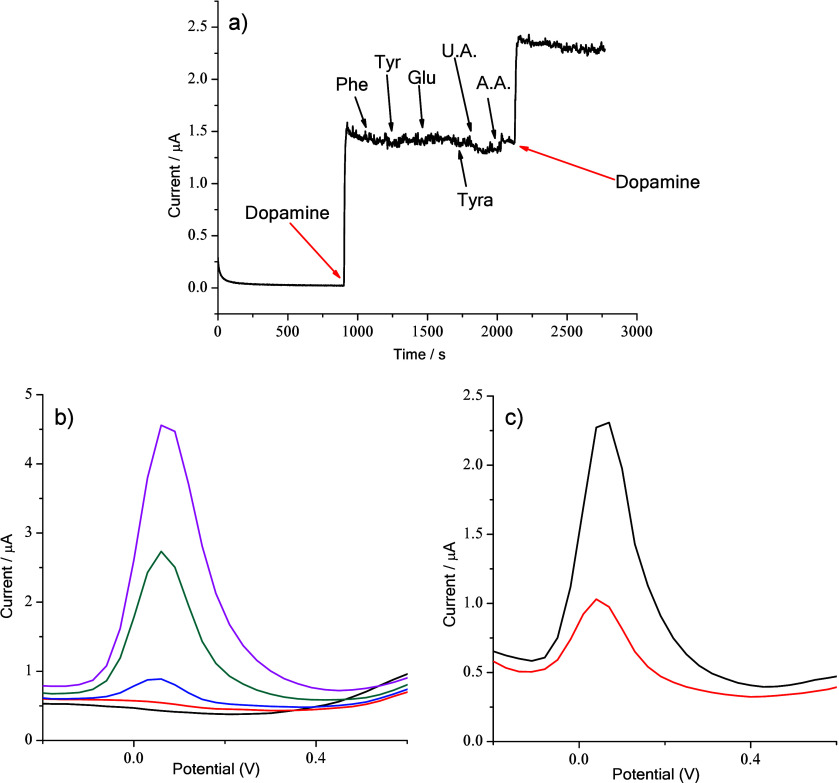
(a) Chronoamperometry response of the HNTK-Cu/SPCE toward dopamine and interferents (200 μM each). (b) DPV analyses to determine 1 (blue line), 7 (dark cyan line), and 15 (magenta line) μM of dopamine, added to PBS 0.1 M (black line) and the FBS (red line). (c) DPV of HNTK-Cu/SPCE in 7.4 PBS and meat samples of chicken (black line) and pork (red line).

To study the sensing behavior of HNTK-Cu/SPCE in real samples, we measured the illegally added dopamine in chicken, pork, and dopamine-free FBS using DPV before and after the addition of 1, 7, and 15 μM dopamine (Figure 8b). The addition of 100 mL of FBS to 3 mL of PBS showed no additional peak apart from an insignificant increase in background current that does not influence DPV measurements (PBS and FBS/PBS are shown as black and red lines). The calculated recoveries ranged from 93.8% to 96.7% (Table 3). This result demonstrates that the sensor can detect the presence of dopamine in real complex matrices.

**Table 3:** Determination of Dopamine Added to FBS with the HNTK-Cu/SPCE Sensor

[dopamine] (μM)	ΔCurrent (μA)	Recovery (%)
1 in 0.1 M PBS	0.48	100 ± 1.2
7 in 0.1 M PBS	2.76	100 ± 0.9
15 in 0.1 M PBS	4.27	100 ± 0.8
1 in FBS	0.45	93.8 ± 2.9
7 in FBS	2.6	94.0 ± 3.5
15 in FBS	4.13	96.7 ± 0.8

Figure 8c shows the current change at the exact expected dopamine oxidation potential (0.3 V), using the already optimized potential step, time pulse, and scan frequency (see Materials), thus indicating the presence of dopamine in the pork and chicken extracts. Using the calibration line shown in Figure 6b and considering the performed dilution (see Materials), we calculated 4.6 and 22.9 mg dopamine/kg of pork and chicken, respectively.^4^ To validate these results, we also performed tests on the extracts for dopamine detection by HPLC analysis, as reported in the literature.^65^ The dopamine values obtained were 4.1 mg/kg for pork and 22.2 mg/kg for chicken, respectively.

The XPS of HNTK-Cu before its use no doubt shows both Cu^2+^ and Cu^0^ (Figure 9a). The tendency of kojic acid itself to oxidize in the presence of light and air is well-known.^66^ In addition, this oxidation is favored by Cu^2+^ ions that are reduced to Cu^+^. However, Cu^+^ is unstable in water (reaction solvent). In fact, by considering the related reduction potential, Cu^+^ ions undergo the disproportionation reaction 2Cu^+^ → Cu^2+^ + Cu^0^ with *E*_0_ = 0.37 V and *K* = 106. Therefore, in aqueous solution, no Cu­(I) can exist, and XPS of HNTK-Cu before its use confirms that our synthetic procedure was effective in obtaining Cu^0^ (Cu 2p_3/2_ at 932.8 eV) and Cu^2+^ (Cu 2p_3/2_ at 935.3 eV) species.^58^ This observation is also substantiated by the UV-DRS measurements that show the presence of a plasmon loss in the range of 600–700 nm due to the Cu^0^ NPs. Therefore, Cu^2+^ ions, partially restored by the above mechanism, can oxidize dopamine to quinone and reduce it to Cu^0^. XPS of the sensor after dopamine adsorption shows the presence of Cu^0^ only (Figure 9b).

**Figure 9:**
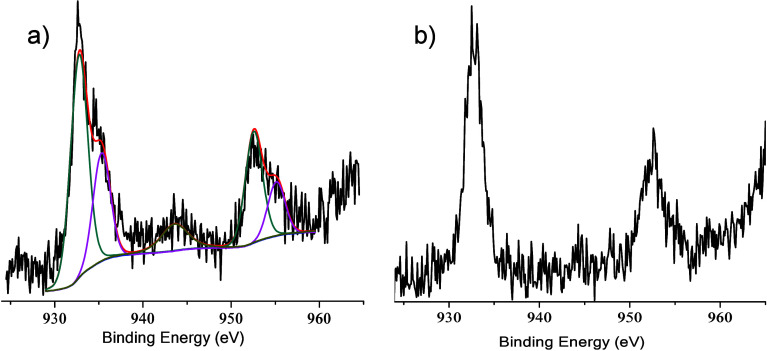
Al Kα excited XPS of HNTK-Cu in the Cu 2p binding energy region, (a) before and (b) after dopamine exposure. The dark cyan, magenta, and dark yellow lines refer to the 932.8–952.6, 935.3–955.1, and 943.6 eV Gaussian components, respectively. The blue line represents the background, and the red line superimposed on the experimental black profile refers to the sum of the Gaussian components.

Based on the results obtained from XPS studies and what is reported in the literature, we propose a possible mechanism of dopamine oxidation/reduction (Figure 10).^33^

**Figure 10:**
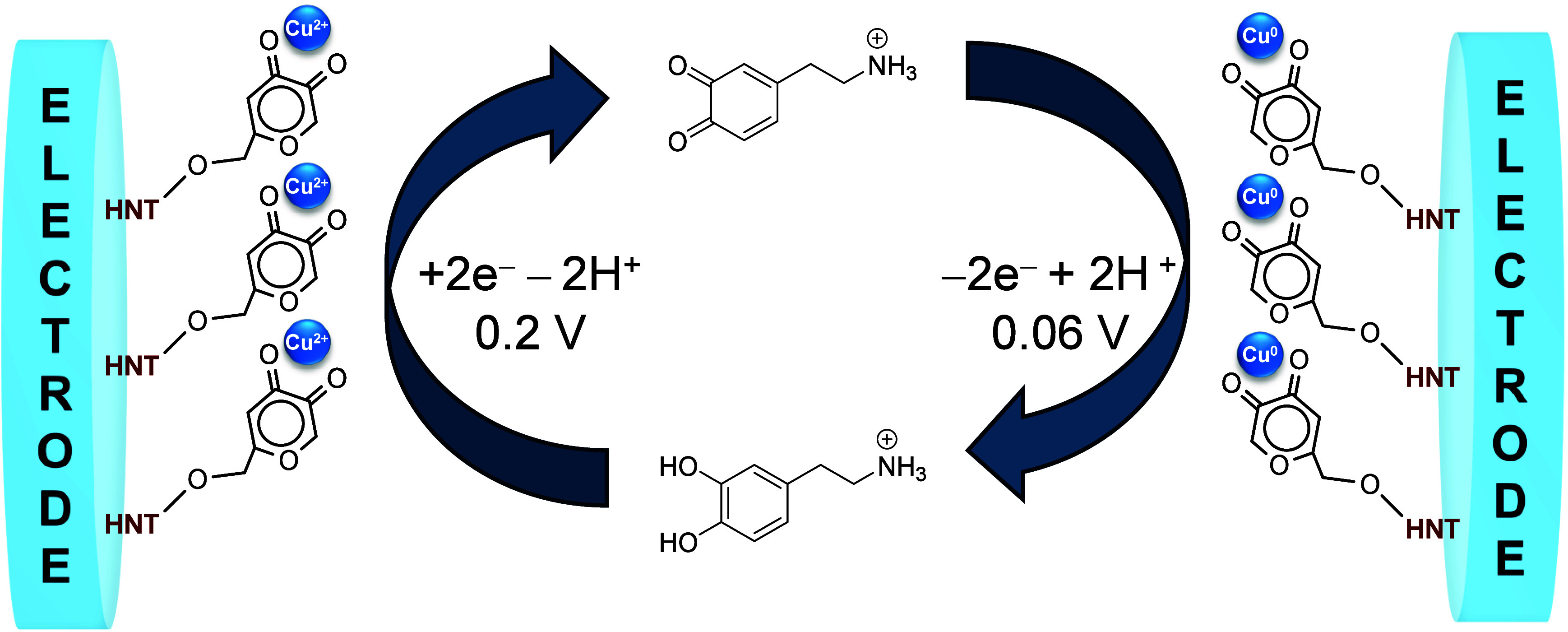
Redox mechanism for the electrochemical response of HNTK-Cu to dopamine.

Table S1 compares the LOD obtained with our eco-nanosensor (HNTK-Cu) to that of some sensors recently reported in the literature, specifically those published in 2024 and 2025. From the comparison, it can be seen that HNTK-Cu possesses the lowest LOD, despite its low Cu content (2.1 wt %). This finding, together with the material’s sustainability and synthesis, makes it an excellent eco-nanosensor for dopamine detection.

## 4 Conclusions

In this study, we successfully developed a sustainable and highly efficient nanosensor for dopamine detection, leveraging the unique properties of halloysite nanotubes and kojic acid. A significant advantage of this approach is its environmentally benign synthesis, employing CPME and water as green solvents to minimize environmental impact. Additionally, the use of naturally derived materials, such as halloysite and kojic acid, enhances the sensor’s sustainability while maintaining excellent electrochemical performance. The eco-sustainability and biocompatibility of our sensor stem from the acclaimed properties of the starting materials HNTs^67,68^ and kojic acid,^69^ the latter of which is, by the way, an FDA-approved molecule. The exceptionally low Cu^2+^ content of 2.1 wt % in the HNTK-Cu complex significantly reduces potential toxicity issues, especially considering that many fertilizers currently on the market are copper-based^70^ and are typically used in much higher quantities than in our sensor. All of this promotes this nanosensor as a safer alternative to conventional dopamine sensors. The HNTK-Cu sensor demonstrated a remarkable limit of detection (LOD) of 68 nM, highlighting its ability to detect even trace amounts of dopamine. Furthermore, it showed excellent sensitivity at 2.165 μA μM^–1^ cm^–2^ and outstanding reproducibility, evidenced by an RSD value of 2.3%, even when challenged with complex real matrices such as pork, chicken, and fetal bovine serum (FBS). Its rapid response, high stability over multiple detection cycles, and cost-effective fabrication collectively position HNTK-Cu as an up-and-coming and sustainable platform for dopamine detection in diverse biomedical and food safety applications.

## Supplementary Material



## Notes

UV-DRS spectra of HNTK, HNTK-Zn, HNTK-Ni and HNTK-Cu, Al Kα excited XPS of the HNTK-Cu sample in the C 1s, O 1s, Al 2p, and Si 2p binding energy regions, ΔCurrent registered in the presence of dopamine (100 μM) at different pH values (3.0–9.0, RSD ≤ 1.8) and at different HNTK-Cu loadings on the SPCE electrode (RSD ≤ 1.5), and comparison of detection performance of HNTK-Cu and detection systems reported in the last two years (2024–2025) for dopamine (PDF)
