# Supplementary material for: Nanosensors Made of Halloysite and Kojic Acid Metal Complexes for Dopamine Detection
Source: ACS Appl Nano Mater. 2025 Jul 31;8(34):16736–47. doi: 10.1021/acsanm.5c02759 (PMC12403182; doi:10.1021/acsanm.5c02759)
Supplement: Supplementary file 1 [file an5c02759_si_001.pdf]

Supporting Information

Nano-Sensors Made of Halloysite and Kojic Acid Metal Complexes for Dopamine Detection

Angelo Ferlazzo [a], Maria Teresa Armeli Iapichino [a], Giulia Calabrese [b], Giovanna D'Accurso [b], Roberto Fiorenza [a], Venerando Pistarà [b], Antonino Gulino [a], Antonio Rescifina [b], Vincenzo Patamia [b,*], Giuseppe Floresta [b,*]

[a] *Department of Chemical Sciences, University of Catania, Viale Andrea Doria 6, 95125 Catania, Italy*

[b] *Department of Drug and Health Sciences, University of Catania, Viale Andrea Doria 6, 95125 Catania, Italy*

*****Corresponding authors*

e-mail: vincenzo.patamia@unict.it, giuseppe.floresta@unict.it

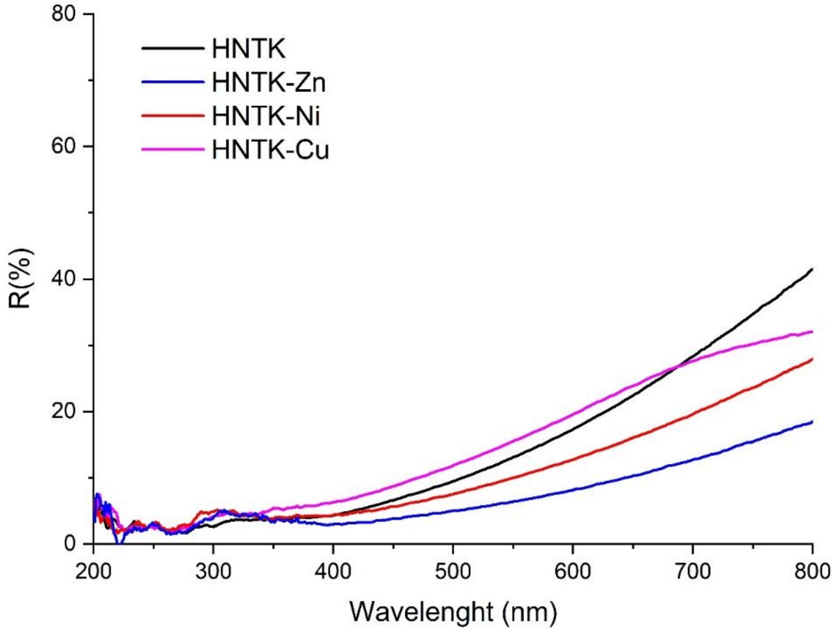

Figure S1. UV-DRS spectra of the examined samples.

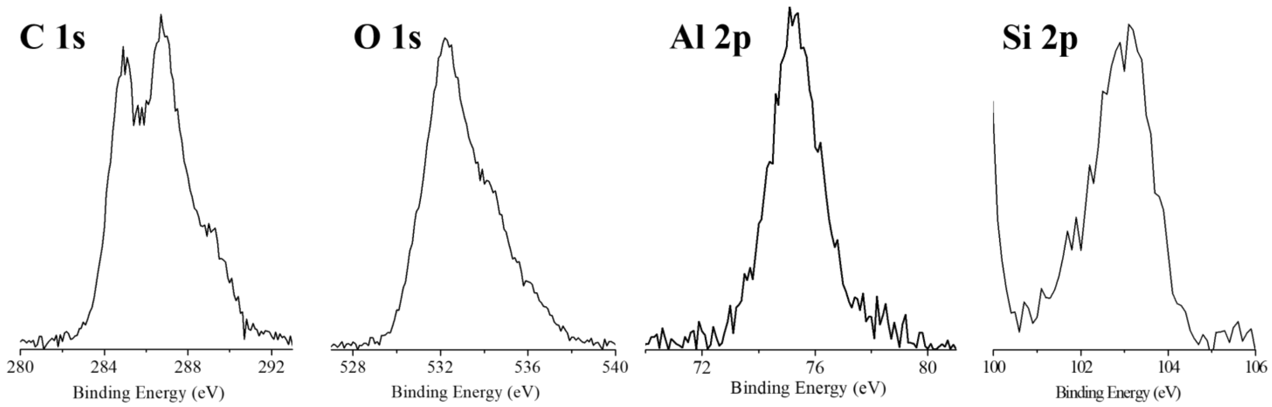

Figure S2. Al Kα excited XPS of the HNTK-Cu sample in the C 1s, O 1s, Al 2p, and Si 2p binding energy regions.

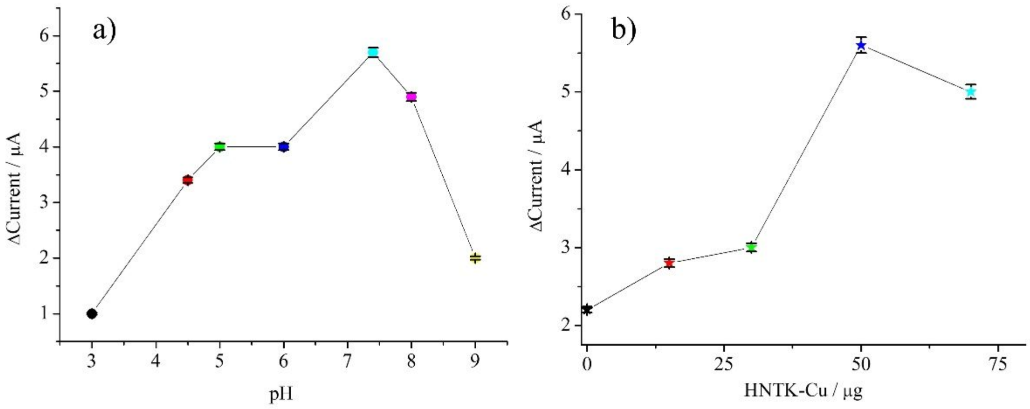

Figure S3. ΔCurrent registered in the presence of dopamine (100 μM): a) at different pH values (3.0–9.0, RSD ≤1.8); b) at different HNTK-Cu loading on the SPCE electrode (RSD ≤1.5).

Table S1. Comparison of Detection Performance of HNTK-Cu and Detection Systems Reported in the Last Two Years (2024–2025) for Dopamine.

**Electrode materials**	**LOD μM**	**Reference**
HNTK-Cu	0.068	This work
Graphite electrode	0.26	[1]
Ni/NiC_x-N-MWCNTs	0.19	[2]
VOG12/GCE	0.29	[3]
PANI:Zn(II)	0.74	[4]
CuAl-LDH	0.33	[5]
CuO-Cu_2O	0.10	[6]
FTO/Ni_2O_3	0.15	[7]
V-CuO/GO/GCE	6.80	[8]
MOFCN	0.13	[9]
CuO/Cu_2O bulk	0.38	[10]
SS-V_2O_5- nitro-phenylene	0.10	[11]
Ag_2CrO_4	1.05	[12]
SPE/MWCNTs-βCD-rGO	0.08	[13]
TiO_2-PVAc-nZVI	1.50	[14]
TM-CNT600/GCE	1.42	[15]

References

(1) Blasques, R. V.; Stefano, J. S.; da Silva, V. A. O. P.; Brazaca, L. C.; Janegitz, B. C. Reusable graphite-based electrochemical sensors for L-dopa and dopamine detection. *Mikrochim. Acta* **2024**, *191(4)*, 197.

(2) Lv, G.; Yang, M.; Pan, Y.; Fan, Y.; Zuo, J.; Liu, X.; Chen, J.; Zhang, S. High-sensitive electrochemical sensor based on Ni/NiCx-integrated functional carbon nanotubes for simultaneous determination of acetaminophen and dopamine. *Colloids Surf. A: Physicochem. Eng. Aspects* **2024**, *703*, 135428.

(3) Brar, L. K.; Ajravat, K.; Kaur, A.; Gupta, A.; Pandey, O. P. VOx@ graphene electro-catalysts for water splitting and dopamine sensing. *Catalysis Today* **2024**, *432*, 114597.

(4) Yıldız, D. E.; Taşaltın, N.; Baytemir, G.; Gürsu, G.; Karakuş, S.; Yıldırım, T.; Şahin, Y. M.; Küçükdeniz, T.; Köse, D. A. Non-enzymatic electrochemical sensors using Polyaniline:Metal orotate nanocomposites for selective dopamine and glucose detection: Predicting sensor performance with machine learning algorithms. *Mat. Sci. Semicon. Proc.* **2025**, *193*, 109492.

(5) Shahparast, S.; Asadpour-Zeynali, K. Development of an efficient electrochemical sensor based on CuAl-LDH using an electrostatic repulsion approach for the selective determination of dopamine in the presence of uric acid and ascorbic acid species. *Electrochem. Commun.* **2024**, *165*, 107756.

(6) Salova, A.; Mahmud, S. F.; Almasoudie, N. K. A.; Mohammed, N.; Albeer, A. A.; Amer, R. F. CuO-Cu_2O nanostructures as a sensitive sensing platform for electrochemical sensing of dopamine, serotonin, acetaminophen, and caffeine substances. *Inorg. Chem. Commun.* **2024**, *161*, 112065.

(7) Mondal, R.; Mukherjee, N.; Ahmed, S. F. Ultrafast, Selective, and ppb Level In Vitro Electrochemical Sensing of Dopamine in a Simulated Interfering Environment: Comparative Study on the Effect of Carrier Type of Electrode Materials. *ACS Appl. Electron. Mater.* **2024**, *6(8)*, 6012–6035.

(8) Bilal, M.; Rehman, Z. U.; Butt, F. K.; Jrar, J. A.; Yang, X.; Zheng, K.; Wang, C.; Hou, J. Enhance electrochemical sensing of ascorbic acid and dopamine using V-CuO/GO nanocomposite. *Colloids Surf. A: Physicochem. Eng. Aspects* **2024**, *703*, 135128.

(9) Yuan, Z.; Zhu, Y.; Wu, H.; Wang, F.; Yin, Y.; Qian, L.; Dai, Y.; Zhang, T.; Xue, S.; Yu, L.; Qiu, F. Metal organic framework modified with carbon nanotube as an electrochemical sensor: Fabrication, excellent stability and sensitive detection of dopamine. *Mikrochim. Acta* **2025**, *208*, 112327.

(10) Mondal, R.; Show, B.; Ahmed, S. F.; Mukherjee, N. Electrochemically selective detection of dopamine over serotonin by CuO/Cu_2O bulk heterostructure electrode. *Bull. Mater. Sci.* **2024**, *47(2)*, 62.

(11) Lemus, S. S.; Lin, J.; Kumar, R.; Keelson, O.; Shringi, A. K.; Yan, F.; Taylor, D. K.; Riaz, U. Nitrophenylene-decorated V_2O_5 nanohybrids: Effect of processing conditions on enhancing the electrochemical performance for oxygen reduction reaction and dopamine sensing. *Mikrochim. Acta* **2025**, *208*, 112453.

(12) Mgenge, L.; Saha, C.; Kumari, P.; Ghosh, S. K.; Singh, H.; Mallick, K. Electrochemical sensing of dopamine using nanostructured silver chromate: Development of an IoT-integrated sensor. *Anal. Biochem.* **2025**, *698*, 115726.

(13) Mwaurah, M. M.; Mathiyarasu, J.; Vinu Mohan, A. M. MWCNTs-Beta-Cyclodextrin-reduced graphene oxide gel based electrochemical sensor for simultaneous detection of dopamine and uric acid in human sweat samples. *Carbohydr. Polym.* **2025**, *350*, 123060.

(14) Qureashi, A.; Nazir, I.; Fatima, K.; Haq, Z.-u.; Ahmad Ganaie, F.; Rafiq, M.; Saleem Khan, R.; Hamid Rather, A.; Bashir, A.; Hussain Pandith, A.; Majeed, S.; Sheikh, F. A. Magnetic

TiO_2-nZVI nanofibers: A highly efficient platform for electrochemical dopamine detection. *Mater. Sci. Eng. B* **2024**, *310*, 117719.

(15) Jaryal, V. B.; Kumar, S.; Singh, D.; Gupta, N. Thiourea-Modified Multiwalled Carbon Nanotubes as Electrochemical Biosensor for Ultra-Precise Detection of Dopamine. *ChemNanoMat* **2024**, *10(6)*, e202300637.
